# Impact of primary care funding on patient satisfaction: a retrospective longitudinal study of English general practice, 2013–2016

**DOI:** 10.3399/bjgp21X714233

**Published:** 2020-12-01

**Authors:** Veline L’Esperance, Hugh Gravelle, Peter Schofield, Mark Ashworth

**Affiliations:** School of Population Health and Environmental Sciences, King’s College London, London.; Centre for Health Economics, University of York, York.; School of Population Health and Environmental Sciences, King’s College London, London.; School of Population Health and Environmental Sciences, King’s College London, London.

**Keywords:** general practice, patient satisfaction, primary care funding, quality of care

## Abstract

**Background:**

Providing high-quality clinical care and good patient experience are priorities for most healthcare systems.

**Aim:**

To understand the relationship between general practice funding and patient-reported experience.

**Design and setting:**

Retrospective longitudinal study of English general practice-level data for the financial years 2013–2014 to 2016–2017.

**Method:**

Data for all general practices in England from the General and Personal Medical Services database were linked to patient experience data from the GP Patient Survey (GPPS). Panel data multivariate regression was used to estimate the impact of general practice funding (current or lagged 1 year) per patient on GPPS-reported patient experience of access, continuity of care, professionalism, and overall satisfaction. Confounding was controlled for by practice, demographic, and GPPS responder characteristics, and for year effects.

**Results:**

Inflation-adjusted mean total annual funding per patient was £133.66 (standard deviation [SD] = £39.46). In all models, higher funding was associated with better patient experience. In the model with lagged funding and practice fixed effects (model 6), a 1 SD increase in funding was associated with increases in scores in the domains of access (1.18%; 95% confidence interval [CI] = 0.89 to 1.47), continuity (0.86%; 95% CI = 0.19 to 1.52), professionalism of GP (0.47%; 95% CI = 0.22 to 0.71), professionalism of nurse (0.51%; 95% CI = 0.24 to 0.77), professionalism of receptionist (0.51%; 95% CI = 0.24 to 0.78), and in overall satisfaction (0.88%; 95% CI = 0.52 to 1.24).

**Conclusion:**

Better-funded general practices were more likely to have higher reported patient experience ratings across a wide range of domains.

## INTRODUCTION

High-quality clinical care and good patient experience are key objectives for most healthcare systems.^1^ Patient experience is a core component of healthcare quality, alongside clinical effectiveness and patient safety.^2^ It is argued that better patient experience contributes to clinical quality improvements by increasing the ability of the healthcare system to respond to patients’ needs, and to identify aspects of poor performance and areas where the organisation might improve.^3^^,^^4^

Primary care is the bedrock of England’s NHS, accounting for nine out of 10 patient contacts in the NHS,^5^ providing the first point of contact and coordinating care in the NHS. There is debate about the adequacy of funding and whether the funding formula perpetuates health inequalities.^6^

The international evidence on the relationship between expenditure and outcomes has mainly focused on US hospital care,^7^^–^^10^ and has produced mixed results when examining primary care.^7^^,^^11^^,^^12^ Two cross-sectional studies have examined the association of funding and quality in English general practices. One found no consistent relationship between funding and patient satisfaction,^13^ and the other found that practices with greater funding were more likely to receive higher-quality ratings from the Care Quality Commission.^14^ This study expands on previous research by using a wider set of patient-reported experience measures and a longitudinal design to examine whether changes in the funding of practices are associated with changes in the experience reported by their patients.

## METHOD

### Study design

Panel data multivariate regression was used to relate general practice funding to patient experience domains over a 4-year period (2013–2014 to 2016–2017) while controlling for patient and practice characteristics.

### Patient experience domains

The GP Patient Survey (GPPS) is a national self-report postal survey of patient experience, which collects approximately 900 000 responses per year from a sample of adults registered at all general practices across England.^15^ To ensure that responses are representative of practice populations, they are weighted to allow for differential response rates across groups defined by age, sex, region, and small-area socioeconomic characteristics.

**Table:** How this fits in

Decisions about the funding of general practice should be informed in part by the relationship between funding and quality. Patient experience is one of three core components of quality in primary care, alongside clinical effectiveness and patient safety. This large-scale longitudinal study of English general practices finds that increases in funding are associated with improvements in reported patient experience of access, continuity of care, and professionalism of practice staff, and with higher overall satisfaction.

The GPPS has questions on patients’ views on access, continuity of care, professionalism of GP, nursing, and reception staff, and overall experience.^11^ See Supplementary Table S1 for a summary of the GPPS questions related to these six domains.^15^ Patient experience scores were calculated as the average of the proportions of patients reporting the top two positive responses (‘good’ and ‘very good’) for questions relating to access (Q3, Q15, Q18, Q25); continuity (Q9); professionalism of GP (Q21), nurse (Q23), and reception staff (Q4); and overall experience (Q28, Q29).

### Practice funding

Three types of funding are considered. The largest is capitation-based (56% of funding in 2016–2017), reflecting factors affecting GP workload including patient age/sex distribution, additional need, morbidity, and list turnover.^16^ These payments are made for the provision of ‘General Medical Services’ [GMS]: the essential services of general practice.^17^ A proportion of practices also receive the Minimum Practice Income Guarantee capitation supplement based on higher historical funding allocations. Performance-related payments including the Quality and Outcomes Framework (QOF) and payments for delivering Enhanced Services^18^ provide 16% of funding. The third category (26%) is payments for operational aspects of general practice, such as appraisal, seniority, information technology, dispensing fees, and postgraduate GP training. Deductions for pensions and professional levies^18^ or payments for premises are not included. The effects of total funding and its three separate components are examined. Funding is measured as pounds (£) per registered patient and adjusted to constant 2016–2017 prices using the Consumer Price Index.^19^

### Patient characteristics

Annual data were obtained from the General and Personal Medical Services database.^20^ Patient characteristics included the proportions of patients aged 0–4 years, ≥75 years, and nursing home residents. Deprivation data for each general practice were attributed as the mean Index of Multiple Deprivation 2015^21^ scores for Lower Layer Super Output Areas (LLSOAs) weighted by the proportion of practice patients resident in each LLSOA. Neighbourhood ethnicity (proportion Asian or black) derived from the 2011 national census was similarly attributed.^22^ The proportion of patients with at least one of 10 long-term conditions was taken from practice QOF data.^23^

### Practice characteristics

Practice characteristics were annual growth in patient list, contract type (Alternative Provider Medical Services, GMS, or Personal Medical Services [PMS]), dispensing status (whether the practice dispenses medication), single-handed practice status (only one GP), and postgraduate training practice status. Practice staffing (GPs, nurses, other staff) was not included as explanatory variables in the model because staffing is likely to be directly affected by practice funding and including these mediating factors may underestimate the full effect of general practice funding.

### Sample

Practice funding and patient experience data were linked for all general practices in England over the 4-year period 2013–2016 (2013: *n* = 7921; 2014: *n* = 7779; 2015: *n* = 7619; 2016: *n* = 7392). A total of 1411 practice-year observations were dropped for practices without a unique reference code in all financial years. Atypical practices with ≤750 registered patients (*n* = 55) or ≤500 patients per full-time equivalent GP (*n* = 46) were excluded following a previously used method.^24^ The use of practice-based demographic data followed a previously used methodology.^25^ Practices with funding in the 1st and 99th centiles were removed from the sample (*n* = 484). The final analysis sample is a balanced panel of 7253 practices followed over the 4-year period. For the analysis of continuity, single-handed practices (*n* = 695) were excluded because the GPPS continuity question asks patients how often they see or speak to the GP they prefer.

### Statistical methods

Linear regression panel models were used to examine the relationship between total general practice funding per patient (measured in SD units) and each of the six measures of patient experience. To reduce the risk of bias from omitted variables correlated with patient experience and with practice funding, the models contain patient and practice characteristic covariates (listed in Table 1) and year effects.

**Table 1.** Characteristics of general practices and their registered populations in England

**Variable**	**2013–2014**	**2014–2015**	**2015–2016**	**2016–2017**

	**Mean (5th, 95th centiles)**			

Practices, *n*	7253	7253	7253	7253

Patients aged 0–4 years, %	6.06 (3.68, 9.16)	5.97 (3.64, 8.95)	5.87 (3.59, 87.74)	5.91 (3.52, 8.56)

Patients aged ≥75 years, %	7.63 (2.49, 12.75)	7.67 (2.50, 12.90)	7.68 (2.51, 12.91)	7.66 (2.46, 13.00)

Index of Multiple Deprivation score	9.22 (0.51, 41.89)	9.22 (0.51, 41.84)	9.23 (0.52, 41.57)	9.25 (0.52, 41.67)

Patients with Asian ethnicity, %	4.02 (0.10, 19.66)	4.02 (0.10, 19.65)	4.03 (0.11, 19.53)	4.04 (0.11, 19.62)

Patients with black ethnicity, %	0.05 (0.00, 19.66)	0.05 (0.00, 14.60)	0.04 (0.00, 13.99)	0.04 (0.00, 13.49)

Nursing home residents, %	0.49 (0.00, 1.47)	0.47 (0.00, 1.46)	0.43 (0.00, 1.40)	0.45 (0.00, 1.35)

Annual growth in patient list size, %	1.34 (−3.79, 7.83)	2.15 (−3.03, 9.15)	2.88 (−3.49, 11.45)	2.70 (−3.21, 10.67)

Patients with ≥1 chronic conditions, %	16.30 (9.24, 24.14)	15.85 (8.86, 24.67)	15.33 (8.57, 23.74)	14.93 (8.29, 23.19)

Contract type, %				
Alternative Provider Medical Services	1.0	3.0	3.1	3.1
General Medical Services	55.5	56.4	62.7	69.3
Personal Medical Services	43.5	40.6	34.2	27.6

Dispensing practices, %	15.0	14.8	14.8	17.8

Single-handed practices, %	9.3	8.9	6.1	6.5

Training practices, %	26.5	25.6	24.8	24.4

In receipt of Minimum Practice Income Guarantee, %	35.6	37.1	40.4	37.8

Both random and fixed practice effect models were estimated. Random-effects models explore the effect of variation in funding both across practices and within practices over time. Fixed-effects models examine the relationship between changes in funding and changes in patient experience over time within practices.

The random-effects model is more efficient than the fixed-effects model but yields inconsistent coefficient estimates unless practice effects are uncorrelated with the time-varying explanatory variables. The Hausman test^26^ was used to test this assumption. Although the null hypothesis of no correlation is rejected (*P*<0.001), random-effects as well as fixed-effects models were used because random-effects models produce estimates of coefficients on variables that do not vary over time or that vary infrequently (such as contract type). Fixed-effects models produce consistent estimates of the coefficients on time-varying variables, such as funding.

It is possible that patient experience reported for a financial year (April to March) depends on funding in the previous year if it takes time to translate funding into decisions that affect patients. Moreover, the GPPS was undertaken part way through financial years (July to September and January to March from 2013–2014 to 2015–2016, January to March in 2016–2017) and some QOF payments are not finalised until after the end of the financial year. Models were therefore compared with current and with a 1-year lag of funding.

Further analyses were undertaken with the three types of funding (capitation, performance-related, and operational) entered as same regression model explanatories. The sample size reduced to 7137 because not all practices engage in performance-related activities.

Robust Huber–White standard errors were used, clustered at practice level to allow for heteroscedasticity. All statistical analysis was undertaken using Stata software (version 14).

### Patient involvement

Funding for this study included funding of a dedicated patient involvement group. Patients were involved in developing plans for the study design, approving the outcome measures, and commenting on the potential impact of outcomes. A lay summary was also provided.

## RESULTS

**Figure 1.**
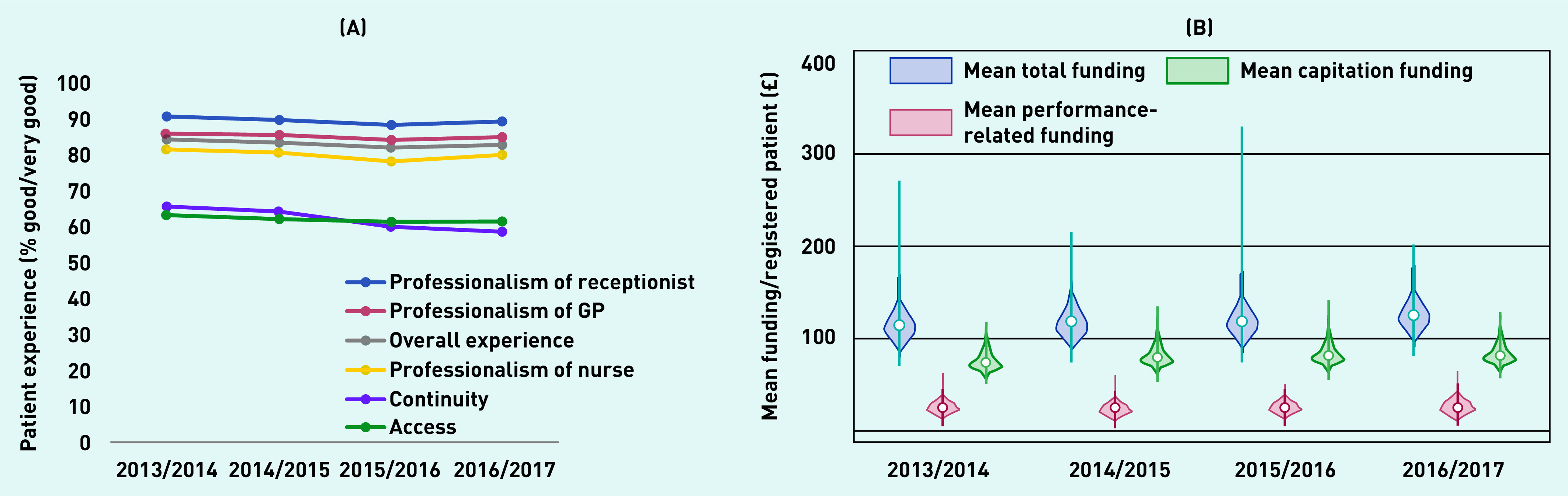
**Patient experience and practice funding 2013–2014 to 2016–2017. A) Patient experience (% good/very good). B) Practice funding per patient, adjusted for inflation at 2016–2017 prices: total funding, capitation funding, and performance-related funding.**

Table 1 gives summary statistics for patient and practice characteristics. Figure 1 shows the changes in patient experience (Figure 1A) and total funding (Figure 1B) from 2013–2014 to 2016–2017. Patient experience worsened slightly in all domains between 2013–2014 and 2016–2017. Total funding per patient increased from £132.42 in 2013–2014 to £137.12 in 2016–2017 (Figure 1B). Total annual funding per patient, capitation funding, performance-related funding, and operational funding across the study period were £133.66 (SD £39.46), £80.89 (SD £12.30), £26.04 (SD £8.89), and £24.64 (SD £33.49), respectively.

**Figure 2.**
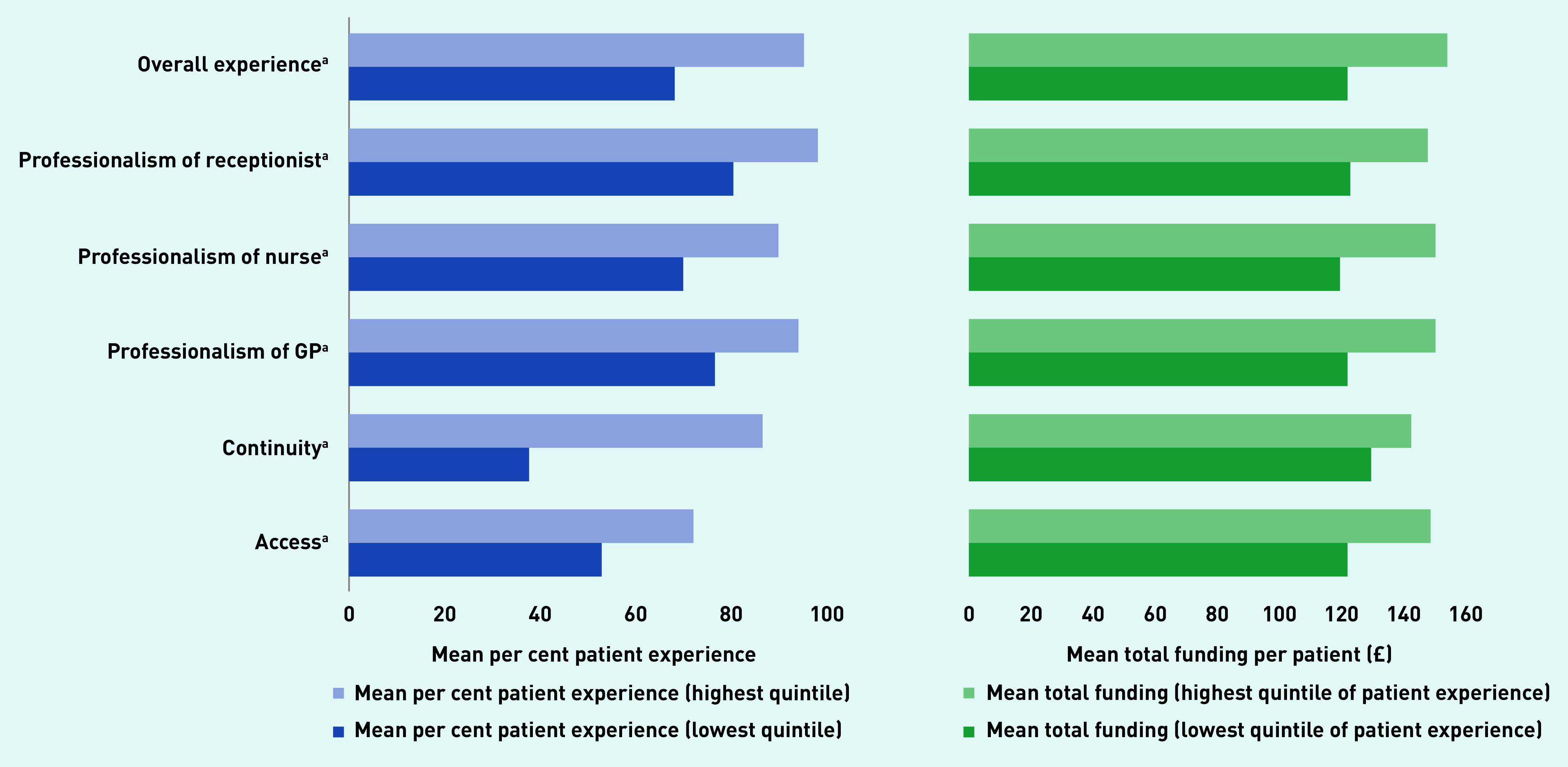
**Practice funding by patient experience: a comparison of funding between practices with the highest and practices with the lowest achieving positive experience quintiles (‘good/very good’).** *^a^***P*****<0.001. Bars illustrate differences in funding. Thus, for ‘overall experience’ the highest achieving quintile had a mean of 94% of surveyed patients reporting positive experience; in these practices mean funding was £154.65. The lowest achieving quintile had 68% positive experience; their mean practice funding was £122.75.***

Figure 2 shows the difference in funding for practices in the highest achieving quintile of patient experience compared with practices in the lowest quintile. In each domain, practices in the lowest quintile received significantly lower mean funding (*P*<0.001, independent group *t*-tests). For example, the mean total funding per patient for practices in the lowest achieving quintile for overall experience was £122.75 compared with £154.65 in the highest achieving quintile (mean overall experience scores are 68% and 94%, respectively).

Table 2 reports estimated changes in overall patient experience (% points) for each 1 SD unit increase in total funding per patient from six models. In all cases the changes are positive and statistically significant. Adding patient characteristics leads to a marked reduction in the funding coefficient (model 2 versus model 1), whereas adding practice characteristics has a much smaller effect (model 3 versus model 2).

**Table 2.** Association of overall patient experience with total funding per patient

	**Model 1**	**Model 2**	**Model 3**	**Model 4**	**Model 5**	**Model 6**
Practices, *n*	7253	7253	7253	7253	7253	7253

Overall patient experience^a,b^	2.40^c^ (2.29 to 2.51)	0.93^c^ (0.77 to 1.08)	0.83^c^ (0.65 to 1.01)	0.89^c^ (0.71 to 1.07)	1.46^c^ (1.27 to 1.65)	0.88^c^ (0.52 to 1.24)

Models contain:						
Year effects	Y	Y	Y	Y	Y	Y
Patient characteristics^d^	N	Y	Y	Y	Y	Y
Practice characteristics^e^	N	N	Y	Y	Y	Y
Practice effects	Random	Random	Random	Random	Random	Fixed

Years	2013/2014 to 2016/2017 2013/2014 to 2016/2017		2013/2014 to 2016/2017	2014/2015 to 2016/2017	2014/2015 to 2016/2017	2014/2015 to 2016/2017

Funding year	Current	Current	Current	Current	1-year lag	1-year lag

a Percentage change associated with 1 SD increase in funding (±95% CI).

b Dependent variable: overall patient experience (Q28, Q29). Calculated from the average of the practice percentages of patients reporting the top two positive responses (‘good’ and ‘very good’).

c P*<0.001.*

d Social deprivation, proportion of patients aged 0–4 years, proportion of patients aged ≥75 years, proportion of patients of black or Asian ethnicity, proportion of nursing home residents, and practice morbidity.

e Contract type, dispensing status, training practice status, and single-handed. NB. All models account for clustering at general practice level. CI = confidence interval. SD = standard deviation.

Comparison of models 4 and 5 shows that funding in the previous year has a larger association with current overall experience than funding in the current year, and model 5 with lagged funding has a better overall fit (see Supplementary Table S2 for details). The Hausman test rejects the assumptions required for the random-effects model 5 to yield consistent estimates (*P*<0.0001). The authors’ preferred specification is model 6, which uses practice fixed effects and a 1-year lag of funding. The fixed-effects model 6 with lagged funding is also better than the fixed-effects model with current funding (see Supplementary Table S2 for details). Model 6 indicates a 1 SD increase in scores in the domains of access (1.18%; 95% confidence interval[CI] = 0.89 to 1.47), continuity (0.86%; 95% CI = 0.19 to 1.52), professionalism of GP (0.47%; 95% CI = 0.22 to 0.71), professionalism of nurse (0.51%; 95% CI = 0.24 to 0.77), professionalism of receptionist (0.51%; 95% CI = 0.24 to 0.78), and in overall satisfaction (0.88%; 95% CI = 0.52 to 1.24).

Table 3 reports the associations between practice funding per patient and six patient experience domains from models with four different specifications of practice effects and funding lags. In all 24 model specifications, funding is positively associated with patient experience and the association is statistically significant in all but two cases (unlagged funding models for continuity).

**Table 3.** Association of patient experience domains with total funding per patient

**Patient experience domain^a,b^**	**Model 3**	**Model 4**	**Model 5**	**Model 6**
Practices, *n*	7253	7253	7253	7253
Access (Q3, Q15, Q18, Q25)	1.06^c^ (0.89 to 1.23)	0.90^c^ (0.69 to 1.10)	1.63^c^ (0.57 to 0.87)	1.18^c^ (0.89 to 1.47)
Continuity (Q9)^d^	0.12 (−0.23 to 0.47)	0.05 (−0.36 to 0.46)	0.98^c^ (0.58 to 1.39)	0.86^e^ (0.19 to 1.52)
Professionalism of GP (Q21)	0.48^c^ (0.35 to 0.61)	0.54^c^ (0.39 to 0.69)	0.82^c^ (0.68 to 0.97)	0.47^c^ (0.22 to 0.71)
Professionalism of nurse (Q23)	0.70^c^ (0.57 to 0.84)	0.72^c^ (0.57 to 0.87)	0.91^c^ (0.76 to 1.05)	0.51^c^ (0.24 to 0.77)
Professionalism of receptionist (Q4)	0.66^c^ (0.53 to 0.78)	0.66^c^ (0.52 to 0.81)	0.86^c^ (0.71 to 1.00)	0.51^c^ (0.24 to 0.78)
Overall experience (Q28, Q29)	0.83^c^ (0.65 to 1.01)	0.89^c^ (0.71 to 1.07)	1.46^c^ (1.27 to 1.65)	0.88^c^ (0.52 to 1.24)
Practice effects	Random	Random	Random	Fixed
Years	2013/2014 to 2016/2017	2014/2015 to 2016/2017	2014/2015 to 2016/2017	2014/2015 to 2016/2017
Funding year	Current	Current	1-year lag	1-year lag

a Results show percentage change associated with 1 SD incr ease in funding (±95% CI).

b All models contain year effects, patient characteristics, and practice characteristics.

c P*<0.001.*

d Continuity model excluded 695 single-handed practices from the analysis. All models account for clustering at general practice level.

e P*<0.01. CI = confidence interval.*

SD = standard deviation.

Table 4 has results from models of overall patient experience in which total funding is replaced by its three components (capitation, performance-related, and operational funding). Although the associations with all three types of funding are positive in all model specifications, they are less precisely estimated than in models using total funding, especially those for capitation funding.

**Table 4.** Association of overall patient experience with types of funding per patient

**Funding variables**	**Model 3**	**Model 4**	**Model 5**	**Model 6**
Practices, *n*	7137	7137	7137	7137
Capitation funding^a,b^	0.02 (−0.12 to 0.17)	0.003 (−0.17 to 0.17)	0.20^c^ (0.06 to 0.34)	0.12 (−0.07 to 0.35)
Performance- related funding ^a,b^	0.36^d^ (0.26 to 0.46)	0.43^d^ (0.32 to 0.54)	0.39^d^ (0.26 to 0.51)	0.18^e^ (0.04 to 0.33)
Operational funding^a,b^	1.10^d^ (0.89 to 1.30)	1.08^d^ (0.86 to 1.32)	1.63^d^ (1.39 to 1.87)	1.10^d^ (0.53 to 1.67)
Practice effects	Random	Random	Random	Fixed
Years	2013/2014 to 2016/2017	2014/2015 to 2016/2017	2014/2015 to 2016/2017	2014/2015 to 2016/2017
Funding year	Current	Current	1 year lag	1 year lag

a Results show percentage change associated with 1 SD increase in funding (±95% CI).

b All models contain year effects, patient characteristics, practice characteristics.

c P*<0.01.*

d P*<0.001.*

e P*<0.05. All models account for clustering at general practice level. CI = confidence interval. SD = standard deviation.*

## DISCUSSION

### Summary

This study found that higher total funding per patient is associated with statistically significant better overall patient experience and with better experience of access, continuity of care, professionalism of GP, nurse, and receptionist. However, the effect is small compared with the overall variation in patient experience. The associations with the previous year’s funding were stronger than with current year’s funding, and for continuity of care the association was only statistically significant for lagged funding. This may be because recent trends to larger practices and shortages of GPs limit the ability of a practice to provide continuity of care.^27^^,^^28^ Patient experience was more weakly associated with capitation funding than with the other two components of total funding (performance funding and operational funding).

### Strengths and limitations

The study dataset covered all general practices in England and included a rich set of practice and patient characteristics to control for factors such as patient ethnicity, age, and sex, which have been identified as potential confounders.^29^ The data provided a 4-year panel of practices and the study was able to examine the relationship between changes in funding for a practice and changes in the experience of its patients. By estimating models with practice fixed effects it was possible to allow for unobserved factors, such as practice style and organisational arrangements, which may affect patient experience,^30^ and which did not change over the time period. However, the fixed-effects specification has the limitation that it cannot produce estimates of such time-invariant factors and its estimates of factors that change slowly will be imprecise. Data were also lacking on some possible confounders, such as the percentage of non-English speakers.^31^

Arguably, the low GPPS response rate (35.7% in 2016)^15^ may lead to imprecise and possibly biased estimates of the association of funding with patient experience. However, using the data that have been weighted by age, sex, and other characteristics, which may affect response rates, will mitigate this type of bias. There is also little evidence that low response rates have introduced bias.^32^ The validity and reliability of the GPPS has also been documented,^33^^,^^34^ and it has been found to have good test–retest properties.^35^ Another limitation in studies of patient experience is the reluctance of patients to comment adversely about consultations.

### Comparison with existing literature

An earlier 1-year cross-sectional study of English general practices found that higher capitation funding was associated with higher reported patient satisfaction in practices with GMS contracts but that there was no relationship in practices with PMS contracts.^13^ The current 4-year longitudinal study has extended these findings to practices with all types of contract and has used other types of funding and a wider set of patient experience measures. Another study used pooled cross-section data and found that higher practice capitation funding is associated with higher Care Quality Commission ratings, including on the ‘responsive and caring’ domain of quality as assessed by on-site practice inspection and consideration of GPPS patient experience scores.^14^

The relationship between patient experience and funding has largely been examined in studies from the US, and results have been mixed. Research using data from the national Medical Expenditure Panel Survey found that patients in the highest quartile of patient satisfaction had 8.8% higher total health spending than patients in the lowest quartile.^8^ Higher hospital and physician spending during the last 6 months of life among Medicare fee-for-service beneficiaries was found to be negatively associated with overall satisfaction but positively associated with satisfaction with interpersonal aspects of care.^9^ Another US study found no significant difference in patient experience of community-dwelling Medicare fee-for-service beneficiaries between the highest and lowest expenditure areas in most categories of patient experience.^10^

### Implications for research and practice

Research methods including the use of coding frameworks for recorded observation of consultations may be an option to tease out the differences between self-reported patient experience and perceptions of observed patient experience.^36^ This was not possible in this study, which used routinely collected, publicly available data.

There is increasing acceptance from UK policymakers that primary care requires an increased share of healthcare funding.^6^^,^^37^ However, there is little information to guide funding decisions. This study provides new evidence that increases in primary care funding may translate into improved patient experience, an important component of healthcare quality. This improvement may not be immediate, and the findings from this study point to a stronger association between investment in practice funding and patient experience after a year. The study contributes to an evaluation of the effect of increased funding and demonstrates that these effects are positive, albeit small. But decisions on general practice funding require consideration of all the effects of funding, including those on health, patient wellbeing, costs in other sectors, and also the opportunity costs associated with increased funding.
